# Impact of Aging and Cytomegalovirus on Immunological Response to Influenza Vaccination and Infection

**DOI:** 10.3389/fimmu.2017.00784

**Published:** 2017-07-17

**Authors:** Shahzma Merani, Graham Pawelec, George A. Kuchel, Janet E. McElhaney

**Affiliations:** ^1^Health Sciences North Research Institute, Sudbury, ON, Canada; ^2^Second Department of Internal Medicine, University of Tübingen Medical Center, Tübingen, Germany; ^3^UConn Center on Aging, UConn Health, Farmington, CT, United States

**Keywords:** cytomegalovirus, influenza, vaccination, infection, older adult, aging, elderly, cytotoxic T-lymphocyte

## Abstract

The number of people over the age of 60 is expected to double by 2050 according to the WHO. This emphasizes the need to ensure optimized resilience to health stressors in late life. In older adults, influenza is one of the leading causes of catastrophic disability (defined as the loss of independence in daily living and self-care activities). Influenza vaccination is generally perceived to be less protective in older adults, with some studies suggesting that the humoral immune response to the vaccine is further impaired in cytomegalovirus (CMV)-seropositive older people. CMV is a β-herpes virus infection that is generally asymptomatic in healthy individuals. The majority of older adults possess serum antibodies against the virus indicating latent infection. Age-related changes in T-cell-mediated immunity are augmented by CMV infection and may be associated with more serious complications of influenza infection. This review focuses on the impact of aging and CMV on immune cell function, the response to influenza infection and vaccination, and how the current understanding of aging and CMV can be used to design a more effective influenza vaccine for older adults. It is anticipated that efforts in this field will address the public health need for improved protection against influenza in older adults, particularly with regard to the serious complications leading to loss of independence.

## Introduction

Cytomegalovirus (CMV) is a β-herpes virus that infects fibroblasts, epithelial, endothelial, stromal, smooth muscle cells, but most importantly, monocytes and dendritic cells (DCs) (1). Depending on the country and its state of development, 25–90% of the worldwide population is CMV seropositive (2, 3) with prevalence higher in older adults (4). Once infected with CMV, the immune system is unable to eliminate the virus, resulting in persistent latent infection. While the contribution of CMV infection to features of immune senescence are well recognized (5, 6), the translation to predicting outcomes in older adults has been much more challenging. Earlier reports of the association between CMV seropositivity and prevalent frailty in community-dwelling older women (7, 8) (frailty determined based on a five component measure: unintentional weight loss, weak grip strength, exhaustion, slow walking speed, and low level of activity) have not always been replicated in more recent longitudinal studies of CMV seropositivity as a predictor of frailty as measured by grip strength (9). However, many other studies have reported an association between CMV seropositivity and frailty (5, 6, 8, 10, 11), and increased mortality (12–15), but these findings are not consistent across all age groups and under all conditions. For example, in the BELFRAIL study, CMV seropositivity was not associated with an increased risk for all-cause mortality in a cohort of very old people. This may have been the result of a survival effect, whereby CMV-seropositive subjects with high anti-CMV titers die at a younger age compared with other individuals. This may reflect CMV reactivation being more common in the end stages of life (15). In terms of the impact of CMV on immune function, CMV seropositivity has been linked to poor CD4^+^ T-cell responses to influenza internal proteins (16), while other studies have found no association between CMV pp65-reactive CD8^+^ T-cells and poor CD8^+^ T-cell responses to influenza internal proteins (17). Although CMV seropositivity in older adults has never been directly correlated with poor vaccine-mediated protection in older adults, high levels of CMV-reactive CD4^+^ T-cells have been associated with an increased risk of viral respiratory illness in elderly nursing home residents (18) and predict increased morbidity and mortality.

Cytomegalovirus and aging of the immune system are associated with oligoclonal expansions of CD8^+^ T-cells, possibly due to an increase in the frequency and magnitude of reactivation of CMV in older compared to young adults (19). At the same time, CMV IgG titers and viral load increase markedly with age (20). These findings suggest that while older adults are able to contain CMV, they do so at the cost of investing ever-increasing resources to control this single pathogen, with the result that immune responses to other challenges may be reduced (21).

Influenza is a single-stranded negative-sense RNA virus that is transmitted through the air by coughing and sneezing and infects epithelial cells, usually in the nose, throat, and lungs. The virus has a major impact on the aging population; ≥90% of annual influenza-related deaths occur in individuals ≥65 years of age (22). Often, influenza itself is not the cause of death, but rather it predisposes older adults to develop secondary bacterial infections and exacerbations of preexisting medical conditions (23, 24). Furthermore, older adults represent the majority of individuals hospitalized with influenza illness (25), which raises concern as hospitalization itself is often followed by a decline in the ability to perform activities of daily living for individuals in this age group (26). Additionally, influenza-related hospitalizations have a significant economic and social impact (27). Although antiviral drugs against influenza are available, vaccination continues to be the most effective method to control infection (28). Prevention of influenza illness through vaccination aids in reducing the burden on the health-care system and maintaining the quality of life of older adults. Hospitalization rates for influenza remain high (25) in spite of evidence that vaccination campaigns can reduce such events (29). Furthermore, multiple impairments associated with CMV and aging appear to lessen the effectiveness of influenza vaccination and reduce the ability to respond to influenza infection to prevent serious complications (30, 31). Recently, it has been shown that influenza vaccination provides good protection against influenza-related hospitalization, but vaccine effectiveness declines as frailty (using the Frailty Index) increases in older adults (32).

## The Role of Antigen-Presenting Cells (APCs) in Influenza Infection and Vaccination

Macrophages and DCs play an important role in directing the immune response to the site of infection. These cells act as APCs and modulate the innate and adaptive immune response. Macrophages initiate the inflammatory responses, while activation of DCs is required for the induction of adaptive immunity.

There are two major categories of macrophages: M1 macrophages, which are induced by Th1 cytokines (IFN-γ and TNF-α); and M2 macrophages, induced by Th2 cytokines (including IL-4 and IL-13). M1 macrophages are characterized by the production of IL-1, IL-12, and TNF-α. In addition, M1 macrophages drive Th1 responses. Aging is characterized by an elevation in baseline inflammatory factors in blood, contributing to a skewed M1/M2 macrophage distribution (32, 33). Specifically, monocytes (macrophage precursors) obtained from older adults prior to influenza vaccination exhibit impaired function with decreased TNF-α and IL-6 secretion, but intact IL-10 responses (34). The dysregulation of IL-10 production from monocytes suggests its potential role in impaired influenza vaccine responses in older adults (34). This dysregulation of TNF-α and IL-6 vs. IL-10 response to influenza vaccination has been linked to the downregulation of the expression of the costimulatory molecules CD80 and CD86 by activated monocytes as a predictor of the antibody response to influenza vaccination (35). A link between CMV infection and dysregulation of DC function may be provided by the finding that CMV itself encodes an IL-10 ortholog, which is known to be expressed during latent infection of myeloid precursor cells (36). CMV-IL-10 inhibits DC function by hindering their maturation and functionality (37) and hence may also play a role in poorer responses to vaccination. Furthermore, plasmacytoid DCs from the elderly are also impaired and produce less TNF-α/IFN-γ in response to TLR7 and TLR9 stimulation (38), which has been associated with poor antibody response to influenza vaccination as well.

## The Role of B-Cells in Influenza Infection and Vaccination

As noted above, CMV may act as an environmental amplifier of immunosenescence resulting in the accumulation of large amounts of late-differentiated CMV-specific effector T-cells (39, 40) and possibly contributing to inflammation. CMV seropositivity is also associated with intrinsically mediated increased levels of inflammatory cytokines in B-cells and diminished B-cell function that predicts poor antibody responses to influenza vaccination (41).

The B-cell response to vaccination decreases with age (42–44) and the compromised effector function of B-cells in the elderly results in lower antibody production and poor Ig class switching (45). While the intrinsic deficits found in B-cells as a result of aging are limited, they are mostly associated with lower levels of long-lived plasmablasts (46–48) and memory B-cells (46, 48, 49). Studies have shown that the age-related decrease in antibody response to influenza vaccination is correlated with extrinsic factors, including impaired T-cell help (39, 40), poor DC function (38), and high IL-10 production by monocytes/macrophages (34) as discussed above.

Influenza vaccines function by generating a B-cell and follicular helper T-cell (T_FH_) response, which in turn results in the proliferation of vaccine antigen-specific B-cells (50, 51). It is believed that IgA and IgM specific for viral hemagglutinin (HA) protect against the establishment of initial infection though neutralization of the virus, while IgG antibodies against nucleoprotein (NP) neutralize the virus if infection becomes established (52, 53).

B-cell defects associated with aging include reduced activation-induced cytidine deaminase (AID). AID is an enzyme required for class switch recombination as well as somatic hypermutation (54) and has been found to correlate with IgG production (55). Prior to vaccination, AID mRNA levels and switched memory B-cell frequencies in response to CpG stimulation correlate with the serum antibody response and are thus predictors of the response to both vaccination and infection. In this context, older adults who are seropositive for CMV show a reduction in both AID and switched memory B-cells relative to CMV seronegatives, and a correspondingly diminished antibody response to influenza A/H1N1 strains.

Another biomarker of B-cell functionality is intracellular TNF-α, which correlates with serum TNF-α levels, which are elevated in the elderly, and more so in CMV seropositives. TNF-α places B-cells in a status of preactivation, which impairs functionality (41). Although the exact mechanism involved in reduced B-cell function by CMV is not known, it may involve a TNF-α feedback loop. Specifically, CMV induces increased production of TNF-α in B-cells *via* NF-κB induction (56). This results in a systemic elevation of TNF-α levels and contributes to CMV-associated B-cell activation, systemic inflammation, and reduced function in these older individuals (41). The important role of TNF-α is further illustrated in B-cell cultures in which TNF-α is neutralized, resulting in improved antibody class switching in elderly individuals (57).

### Ambiguity of the Role of CMV and Aging on the Antibody Response to Influenza Vaccination

Some reports indicate that CMV seropositivity may be associated with better antibody response to vaccination in younger adults (58). This is different in studies involving older adults, where CMV seropositivity has been variably found to be associated with beneficial (59), negative (41, 58–62), or negligible effects (58, 63). The overall impact of CMV infection on influenza vaccine responsiveness remains controversial, as it is depends on many variables. Different studies performed with different seasonal vaccines, tested in different populations, at different times, are difficult to compare directly. As such, there have been no studies that have directly linked CMV seropositivity with increased risk of influenza illness in vaccinated older adults.

In addition to lack of consensus on the impact of aging and CMV seropositivity on antibody responses to influenza vaccination, investigating this issue is further complicated by differing responses to strains of the virus. Vaccine efficacy in the elderly against H3N2 is particularly poor compared to H1N1 or B strains (64, 65). One explanation for an apparent lack of responsiveness in the elderly may reside in the manner in which antibody responses are quantified, which is dependent on the immunological history of the individual. Thus, older adults who already have a high-antibody titer prior to vaccination may be classified as non-responders if they do not further increase an already-protective titer. More importantly, comparisons of the antibody response to influenza vaccination in young and older adults have been confounded by the effects of age and exposure history related to prior vaccination (66). In addition, these differing observations may be explained in the context of original antigenic sin, which supports the notion that vaccination re-stimulates immunological memory of past exposure to a similar strain, and may explain the relative protection of older adults against the pandemic H1N1 (pH1N1) strains (67). The theory of vaccine re-stimulation has not been explored in the context of CMV, but highlights the importance of identifying which subtype of influenza is being studied and a consensus as regards to the definition of vaccine “responder.”

Contradictory observations of influenza strain-specific titers post-vaccination between CMV-seropositive and -negative individuals have also been identified. Specifically, CMV^+^ subjects were found to have higher antibody titer to H1N1 (58, 59, 61), while others have observed the opposite (41). Similarly, in some studies, no association was observed between CMV status and H3N2-directed antibodies (63), while others have reported lower H3N2 antibody responses in such subjects (62). Those identifying an improved response to vaccination have hypothesized that CMV infection is accompanied by a higher level of a low-grade chronic inflammation that in turn provides an ongoing stimulation to the immune system in older (68) and younger adults (58).

It should be noted that studies in this area have used different measures of the antibody response to vaccination as a correlate of protection. Specifically, some have reported peak antibody response, while others measured antibody persistence. Although peak antibody titers after vaccination depend mainly on short-lived plasma B-cells, antibody persistence depends on memory B-cells and long-lived plasma cells. As such, antibody persistence may be a more meaningful measure of clinical protection. Some apparent discrepancies in the literature could derive from such different measures.

Other possible reasons for the discrepancies reported in the literature may be related to confounding factors such as medications, as illustrated in a recent study by Reed et al. These investigators identified a poorer antibody response (quantified based on antibody persistence) to vaccination in CMV-seropositive older adults, but only if they were taking β-adrenergic-blocking drugs (69). β-adrenergic blockers, a class of drug commonly used for blood pressure control, may also influence immune responses (70) and could therefore represent a confounding factor resulting in non-consensus of previous studies of antibody responses in older adults. However, it should also be noted that the use of β-blockers may simply reflect non-specifically generally poorer health or could represent an association with other health conditions relating to immunosenescence. The potential impact of drug treatment on vaccine response is further illustrated in the case of statins and antibody responses to influenza vaccination in older adults (71) as well as vaccine efficacy (72). Statins are known to influence immune responses *via* multiple different mechanisms (73), indicating a need to investigate the relationship further, especially since this class of drug is used by a growing number of older adults (74, 75).

### Molecular Genetics As a Tool to Investigate B-Cell Function in the Context of Aging and CMV

Sequencing of the immunoglobulin heavy chain has been conducted to study B-cell receptor (BCR) repertories. It was found that V (variable), D (diversity), and J (joining) usage is consistent between age groups, although mutations in V genes are associated with CMV seropositivity. Furthermore, mutations in IgM and IgG sequences are higher in the elderly, and more so in those who are CMV seropositive (76). This suggests that repeated antigen exposure with aging and CMV reactivation induces B-cell proliferation and IgG gene mutations.

In a groundbreaking study, de Bourcy et al. used next-generation sequencing technology to study BCR diversity. They showed that while BCR repertoires become more specialized over the lifespan, they also demonstrate decreased capacity for plasticity or adaptability. Relative to the young, older adults have a smaller naïve repertoire and lower intra-lineage diversity, resulting in a reduced ability to mount a diverse response to novel antigens (77). This suggests that annual updates of the strains of influenza contained in the vaccine are less likely to induce antibody responses to new viral variants in older adults.

The association of leukocyte telomere shortening with some aspects of aging has been well documented. In a seminal study of the correlation between telomere length of B-cells and humoral immune responses to influenza vaccination in adults over the age of 70, it was shown that those with longer telomeres (6.3 kb) had superior antibody responses (based on fold-increase of influenza-specific antibody titers) relative to those with shorter telomeres (5.6 kb) (17). While the mechanism involved is still not well understood, telomere length could be used as a marker for immune function and vaccine responsiveness.

## The Role of T-Cells in Influenza Infection and Vaccination

The antibody response to influenza virus plays a vital role in protection against influenza infection, but epitope-specific T-cells are also critical (78–80). Although assessment of antibody responses to influenza vaccines is mainly used as a measure of efficacy, studies continue to show that humoral immunity by itself does not provide sterilizing immunity against infection in older adults, and that T-cell responses are critically important when antibody-mediated protection fails (81). Furthermore, T-cell responses are cross-reactive within the strains of influenza A or influenza B, allowing for broad protection against drifted strains of influenza (82).

Decreased output of naïve T-cells resulting from thymic involution after puberty results in a reduced ability to respond to novel antigens (83) and has been linked to poor response to influenza vaccination in the elderly (84). Studies have shown that telomere length of T-cells specific for CMV are longer on average than those specific for influenza and may suggest that CMV continues to recruit cells from the naïve T-cell pool over time (85).

Aging is correlated with a loss of naïve CD8^+^ T-cells, more so than naïve CD4^+^ T-cells. This loss in naïve cells is not associated with CMV seropositivity (86). The loss of naïve CD4^+^ T-cells is associated with an increase in effector and effector memory CD4^+^ T-cells and is observed essentially only in CMV-seropositive individuals (86). These findings illustrate the distinct, and sometimes additive effect of aging and CMV in different T-cell populations.

### Helper T-Cells

Influenza infection induces HA-specific CD4^+^ T-helper cells (87), resulting in a diverse antibody response (88). Th2 responses stimulate antibody production that is driven by the production of cytokines, including IL-4, IL-5, IL-10, IL-13, IL-31, and IL-33 by mast cells and eosinophils, which are responsible for a Th2 response, thereby leading to the activation of B-cell clones and production of influenza-specific IgG1 and IgE (89, 90). The regulatory T-cell (Treg) response results in the expression of IL-10 and TGF-β, which further hinders a Th1 response (91).

Aging is associated with an increasing acquisition of a Th2 bias, specifically with an increase in CD4^+^CD294^+^ (Th2) cells (92). Furthermore, some studies have reported a decline in the total number of CD8^+^ T-cells and increases in T-helper cells reflected in a lower Th1:Th2 ratio (92). IL-10 and other cytokines produced by Th2 or Treg have been associated with reduced cytotoxic T-lymphocyte (CTL) activity in older adults (93) and against *ex vivo* influenza virus challenge (94). Although Th2-associated cytokines do not help in the recovery from influenza infection (95), these cytokines continue to be expressed at high levels at the site of influenza infection and may be a factor in inflammation and lung damage associated with infection (78).

#### Role of T_FH_ Cells during Influenza Infection and Vaccination in the Elderly

IL-12 production by activated DCs induces naïve CD4^+^ T-cells to differentiate into IL-21-producing T_FH_ cells (96, 97). Elevated IL-21 is positively correlated with CMV seropositivity (98) as it is believed to be required for the maintenance of latent infection (99–101) as well as clearance of acute viral infections (102, 103).

T_FH_ cells are a separate type of helper T-cells that are known to promote germinal center formation, B-cell survival, proliferation, class switching, plasma cell differentiation, and somatic hypermutation (104–108) and are hence found in germinal centers (GC T_FH_) and in peripheral blood (pT_FH_). Studies have found a direct relationship between the frequency of activated pT_FH_ cells following vaccination and influenza vaccine-induced antibody responses (109–111). It has also been shown that pT_FH_ cells isolated post-influenza vaccination are better able than other CD4^+^ T-cell subsets to support B-cell differentiation and to stimulate influenza-specific antibody secretion (110).

A study of older women found a high frequency of activated T_FH_ cells (CD38^+^HLA-DR^+^Ki-67^+^). The presence of activated pT_FH_ cells in older women prior to influenza vaccination was negatively correlated with antibody titers post-vaccination and suggests that activated pT_FH_ cells are less capable of providing help to B-cells when faced with new antigens (112).

### Cytotoxic T-Lymphocytes

Aging creates specific challenges to effective CTL activity against infection: (a) T-cell receptor (TCR) diversity reduction, (b) reduced effector function of cells, (c) cell type frequency changes, and (d) general inflammation.

Cytomegalovirus elicits CD4^+^ and CD8^+^ T-cell responses (113, 114), which play an important role in maintaining latency of CMV infection (115). The immune response necessary to maintain latency has two major consequences: driving T-cells to a late-differentiated state associated with immunosenescence (6, 14, 116) and memory inflation (117). The latter develops from chronic antigen exposure as a result of CMV infection (118, 119), but interestingly Epstein–Barr virus does not have the same impact on inflation (120). CMV-specific memory T-cells account for 0.1–40% of the total memory population in the periphery (113, 114). The high frequency of CMV-specific T-cells develop during the first year after infection and either gradually increases or remains constant long term (121–123). Over a lifetime, a large group of T-cells recognizing CMV epitopes emerges, the majority of which may be dysfunctional (124). This contributes to the concept of a restricted “immunological space,” whereby the T-cell populations consisting of dysfunctional clonally expanded and anergic cells are targeted toward a small number of epitopes (125). In addition, aging results in a decrease of the TCR repertoire, which is associated with a poor response to influenza vaccination (126–129). Furthermore, the repertoire may become oligoclonal due to extended lifespan and homeostatic turnover of naïve T-cells (130).

On infection, viral epitopes bind to the major histocompatibility complex (MHC) molecules of APCs, and through the interaction with TCRs activate naïve or memory T-cells to become effector CTLs (131, 132). The majority of the CD8^+^ T-cell epitopes derived from influenza virus are contained within the NP and matrix 1 (M1) proteins. As a result of the homology of these internal proteins and highly conserved epitopes across the different subtypes (A/H1N1 and A/H3N2), the CD8^+^ T-cell response to influenza is cross-reactive among all of the strains of influenza A. Activation of T-cells leads to their migration to the infection site where they recognize influenza virus-infected cells and eliminate them *via* lytic activity. While CTL killing of influenza-infected cells can be mediated through Fas- (132), and TRAIL- (133) associated pathways, the dominant mechanism appears to be perforin-mediated killing (134). Recent studies suggest that perforin-mediated killing through granzyme B (GrB) apoptotic pathways is the most critical for viral clearance (135).

#### Role of GrB in Protection against Influenza Infection

A correlation between low GrB prior to H3N2 infection, fever, and lack of seroconversion is indicative of the association of cell-mediated immunity and illness severity (136). GrB levels increase in response to H3N2 infection independently of serological responses (136), with a deficiency in the production of GrB and IFN-γ in CD8^+^ T-cells observed in vaccinated older adults (137, 138).

Granzyme B has been associated with clinical protection from influenza (139) and is produced by both CD4^+^ and CD8^+^ T-cells (140). Late-differentiated T-cells (CD45RA^+^GrB^+^Perforin^−^) particularly CD8^+^ subsets are abundant (with as many as 50% of these cells producing GrB in the resting state) and are associated with poor CD8^+^ T-cell cytolytic activity following influenza vaccination (137, 141). Of note, the cytolytic activity of CD8^+^ T-cells dramatically declines by 10 weeks post-vaccination, and while this occurs to a lesser degree in CD4^+^ T-cells, their cytolytic potential is relatively minor compared to CD8^+^ T-cells (137). However, this discordant change in CD4^+^ and CD8^+^ vaccine-specific T-cells suggests that CD4^+^ CTLs in older adults could be targeted to promote cell-mediated immune protection *via* vaccination.

CD4^+^ T-cells in the lung expressing GrB and perforin have cytolytic activity against influenza in mice (142, 143). Using influenza M1 peptide stimulation, it has been shown that the proportion of subjects mounting a CD4^+^ T-cell response was lower in CMV-seropositive than seronegative individuals (16).

Granzyme B and perforin-expressing CD4^+^ T-cells also produce IFN-γ, suggesting a Th1 lineage (143, 144). Many CD4^+^ T-cells responding to influenza in the lung produce IL-10, largely in cells also producing IFN-γ (78, 145). This IL-10 production by influenza-specific CD4^+^ T-cells results in reduced protection (146) by suppressing cytokine production in Th17 cells (78, 145), but also plays an important role in limiting immunopathology (78).

Cytomegalovirus-seropositive older adults have higher levels of GrB in resting T-cells, the majority of which have a late-differentiated T-cell phenotype (CD45RA^+^) or are CD28^−^ (147). The accumulation of GrB in putatively terminally differentiated CD8^+^ T-cells in the absence of perforin *in vivo* (147) suggests that GrB may be released into and accumulate in the extracellular space, resulting in inflammation and tissue damage (148–150). It has also been shown that *ex vivo* live influenza virus challenge results in a lower GrB response in CMV-seropositive compared to -seronegative older adults (63), further suggesting an impairment of CTL response to influenza mediated by CMV.

#### Impact of Reduced CD28^+^ T-Cells in Elderly and CMV Seropositivity

CD28 has an important function as a costimulator in the activation of T-cells and influences their susceptibility to apoptosis (151). It is required for optimal T-cell activation, but its level of expression by CD8^+^ T-cells decreases with age (152, 153). This cell phenotype has been associated with a poor response to influenza vaccination (39, 40, 154), and it has been suggested that it has some similarities with replicative senescence (155). It has also been shown that CMV infection contributes to the accumulation of these cells (156, 157). In addition to CD8^+^CD28^−^ cells impacting vaccine response, late-stage differentiated CD4^+^ T-cells, lacking CCR7, CD27, and CD28 and re-expressing CD45RA are also found in CMV-seropositive subjects and correlated with poor vaccination response (158). A low frequency of CD45RA re-expressing late-differentiated CD4^+^ T-cells are found in CMV-seropositive individuals, independent of age (159–161), with the majority of these cells being CMV specific (158, 160).

#### CD4:CD8 Ratio As a Biomarker of CTL Function

CD4:CD8 T-cell ratios contribute to immune risk profiles with a ratio of less than 1 being predictive of 2-year mortality in some studies (162, 163). CMV infection has been found to be associated with a CD4:CD8 of <1 (13, 164). These findings are consistent with the notion that the clonal expansions of CD8^+^ T-cells observed in the elderly (165–167) are to a large extent CMV-specific (124) and associated with mortality (168). Changes in the ratio were found to be the result of an increase in CD8^+^ T-cell populations, specifically, CD27^−^, CD28^−^, CD56^+^, and CD57^+^, CD45RA^+^, and CD45RA^+^/RO^+^ cells (13), markers that indicate reduced effector functionality and provides support for the finding of poor humoral response to influenza vaccination in those with a low CD4:CD8 ratio (169). Furthermore, the majority of the CMV-specific CD8^+^ T-cells in the elderly have reduced functionality, supported by the finding that the fraction of cells producing IFN-γ in response to peptide stimulation in the elderly was significantly lower than in the young (124). Elderly with a CD4:CD8 ratio <1 had about 10% of total CD8^+^ T-cells specific for a single CMV epitope (170), but in the group with a CD4:CD8 ratio >1, the frequency was similar to the middle-aged group (124).

## Alterations to Vaccine Design

In addition to the aging immune system and CMV seropositivity both potentially hindering the immune response to influenza vaccination, other challenges to effective vaccine design are also in play. Challenges faced particularly in influenza vaccine development lie in the high level of strain divergence from season to season resulting from error-prone replication of the influenza virus *via* RNA-dependent RNA polymerase, recombination, and genetic drift (171). While the plasticity of the virus is sustained when variability accumulates in the HA and neuraminidase (NA) proteins, internal proteins are not as capable of maintaining functionality in the face of mutation accumulation, resulting in much less variation in the matrix and NP (172). The current vaccine strategy is designed to stimulate the development of antibodies against the HA and NA proteins, resulting in the ongoing need for annual vaccine modification to account for viral strain variation, which may be obviated to some extent through vaccines designed to also target internal proteins.

While antibody titers are the generally accepted standard for the testing of influenza vaccination protection, it has been suggested that it is a poor measure if used alone to assess protection in the elderly (173). The limitations of antibody titers are apparent when examining the strain-specific differences in the elderly. For example, vaccine efficacy against H3N2 in the elderly is particularly poor compared to H1N1 or B strains (64, 65), while concurrently, others have found higher antibody titers against the H3N2 strain post-vaccination in this group (174–176).

The focus on humoral immune protection against influenza has its limitations, as circulating strains may not match the vaccine (177). Current vaccines do not induce sufficient cross-reactive CD8^+^ T-cells to provide protection against non-homologous influenza A virus challenge (178), but this would be an advantageous characteristic of future vaccine candidates.

### Dosage

Studies comparing dosage of trivalent inactivated influenza virus vaccines in older adults have found that people who received high dose vaccinations had significantly higher antibody titers for all three strains (to varying degrees) than those who received standard dose vaccinations (179, 180). High dose vaccine recipients had a greater frequency of pT_FH_ cells post-vaccination than those receiving a standard dose. Specifically, the expression of CD278 (also referred to as inducible T-cell costimulator: ICOS) on the pT_FH_ cells was elevated (181), suggesting an increase in their ability to provide B-cell help. Furthermore, as mentioned previously, the frequency of pT_FH_ cells was a predictor of seroconversion for all three vaccine strains (181). It has also been shown that the longevity of the antibody response is not influenced by vaccine dose (182). An assessment of the impact of vaccine dose on the cellular immune response has been limited to analysis of IFN-γ response, which found no significant difference (182, 183). However, this important issue requires further study.

While the high dose vaccine has been shown to deliver better clinical protection in older adults (184), further investigation as to the mechanism is required. Furthermore, the impact of vaccine dose in more vulnerable older adults, including those who are CMV seropositive also requires investigation.

### Adjuvants

The elderly might benefit from the increased application of adjuvanted vaccines. For example, glucopyranosyl lipid adjuvant (GLA) is a TLR4 agonist that has been found to be safe and well tolerated (185). GLA, when combined with Fluzone, a split virus vaccine, showed enhanced antibody response as well as a shift to a Th1 cytokine profile in mice (185). *In vivo*, GLA produces a shift toward a Th1 cell-mediated response to influenza challenge by reducing IL-10 expression along with an increase in GrB activity (186). It has also been shown *in vitro* that GLA can induce the maturation of human DCs with an associated release of Th1-inducing cytokine and chemokine constellation (187).

Older adults seropositive for CMV have been found to have lower levels of activated DCs than young CMV^+^ adults, as it is believed that the older group has a greater control of infection due to higher CMV-specific antibodies. Due to a lower preactivated state, the DCs of older adults, regardless of CMV status, were also relatively more responsive to TLR4 antagonist (in this case LPS) and were able to produce TNF and IL-6 at the same level, and same cell frequency as younger adults who were CMV seronegative (188). Thus, the use of a TLR4 agonist in influenza vaccines for the CMV-seropositive elderly may be a potential adjuvant to improve vaccine efficacy in this group.

### Vaccine Antigen

Conserved proteins, NP and M1, share high levels of homology among influenza strains (189, 190) and contain immunodominant MHC Class I and II epitopes (191). At the present time, influenza vaccines are designed to have specific quantities of HA protein, but the quantity of the internal proteins are believed to be low and are not quantified as part of vaccine formulation quality control (192). Evidence suggests that the inclusion of NP and M1 proteins in influenza vaccines would develop a Th1 memory and provide more effective protection in older adults.

Clinical studies of mRNA-based vaccines demonstrated their ability to elicit functional antibodies and T-cell responses (193, 194). Unlike previously considered DNA-based vaccines, there is no concern regarding genome integration, or the need to design nuclear transport mechanisms for mRNA vaccines. RNA-based vaccines allow for cell entry, viral protein translation, and broad immune responses, while also acting as an adjuvant (TLR7/8) (195). Two different forms of RNA-based vaccines are currently being developed against influenza: non-amplifying mRNA (196) and self-amplifying mRNA molecules (197, 198). A modified vaccinia virus Ankara-based vaccine against influenza has been developed which consists of non-replicating RNA encoding both NP and M1 proteins (199). This vaccine was found to be safe and able to stimulate T-cell responses in older adults similar in magnitude to young adults (200). Amplifying mRNA (SAM1 technology) is based on non-viral delivery of antigen-encoding RNA by modified alphavirus single-stranded RNA, which allows for viral RNA replication and greater viral protein translation (193, 194, 197). In a comparison of replicating and non-replicating vaccines, it was found that self-replicating vaccines elicit significantly stronger cellular and humoral immune responses, which was suggested to be the result of greater antigen presentation (201). These findings suggest that self-replicating influenza RNA vaccines may be used to overcome the effects of immune senescence and CMV seropositivity.

### Vaccination Schedule

A study mapping the prevalence of influenza-specific antibodies in children found that by the age of 6, all children had seroconverted and thus had immunological memory for at least one influenza virus strain (202). Over time, this memory is boosted and diversified by subsequent infections with drifted influenza virus strains. In turn, this memory response (both humoral and cellular) can act to protect against infection with similar stain variants, referred to as cross-protection. Cross-protection was shown in the pH1N1 virus identified during the 2009–2010 and 2013–2014 influenza outbreaks which resulted in high rates of morbidity and mortality. Analysis of the CD8^+^ T-cells specific for the 2009 pH1N1 virus identified epitopes shared with the 1918 pH1N1 strain, as well as stains circulating prior to 1945 (203). These findings corroborate the observation that the severity of the influenza illness in the over-65 age group infected with pH1N1 was considerably lower than other influenza seasons (204), suggesting cross-protection. Furthermore, it was found that the majority of severe cases of pH1N1 infection occurred in young adults (205). This has led to suggestions that vaccines may only be effective due to their ability to act as a booster to memory CD8^+^ T-cells remaining from previous infections, rather than creating a memory response to new targets (137). Furthermore, evidence suggests that T-cells generated in youth can remain protective over decades (206, 207), while those derived later in life are severely impaired (208, 209). Thus, it could be postulated that the most effective way to ensure protection *via* vaccination in older adults would be to design a vaccine strategy targeted at youth.

Vaccine effectiveness against influenza declines with time after vaccination over the winter season and is most evident in older adults, with efficacy lasting 140 days for H3N2 and 200 days for influenza B (210). Interestingly, vaccine effectiveness appeared to be maintained for over 200 days for subtype H1N1 (210). Influenza vaccines are only administered once a year but the impact of a double dose or booster vaccines is being studied to determine whether efficacy and level of protection can be improved. At present, a clinical trial of vaccines administered bi-yearly in older adults is underway (http://ClinicalTrials.gov: NCT02655874). The benefit of two-dose influenza vaccines has been shown under somewhat different circumstances in studies of solid organ transplant recipients where a second influenza vaccine dose after 5 weeks resulted in higher rates of seroconversion and seroprotection (defined as titer ≥1:40) (211). Frail older adults who were non-responders to an initial dose of influenza vaccine showed a decline in antibody titers to the A/H3N2 strain following a booster dose of influenza vaccine and derived no clinical benefit from this booster strategy (212). These results are consistent with our earlier studies showing a significant IL-10 response to a booster dose vaccination and a decline in the antibody and GrB response to influenza challenge following vaccination, compared to those older adults who received a single dose of influenza vaccine (213). Studies of H5N1 have shown that a two-dose approach using heterosubtypic H5 antigen results in a greater magnitude of T-cell cytokine response with heterosubtypic protection, but this does not seem to be the case in older adults (214). As such, the impact of a two-dose influenza vaccine is not yet clear and requires further investigation to determine effectiveness.

### Reducing the Impact of Immunosenescence through Anti-CMV Strategies

One possible method to improve influenza vaccine efficacy might be an anti-CMV strategy *via* vaccination or treatment. While the majority of CMV treatment strategies have been developed for the prevention of congenital CMV infection or for the immunocompromised, there is potential for its application in the wider population.

The challenge in CMV vaccine design lays in the extensive genetic diversity of CMV strains due to recombination (215, 216). Several multi-component subunit (217), recombinant live-attenuated (218), and DNA (219) vaccine candidates aimed at achieving broad cross-neutralizing humoral and cellular immune responses are currently under investigation. One clear caveat for the CMV vaccine strategy to be effective is the need for its administration at a young age, and the ability for the vaccine to impart long-lasting immunity. Drug treatments may be a more practical approach in the short-term including those with anti-CMV activity *in vitro* currently being tested in clinical trials: a protein kinase inhibitor with specific activity against CMV (220); a CMV terminase inhibitor (221); and a broad spectrum antiviral agent (220).

## Conclusion

**Figure 1:**
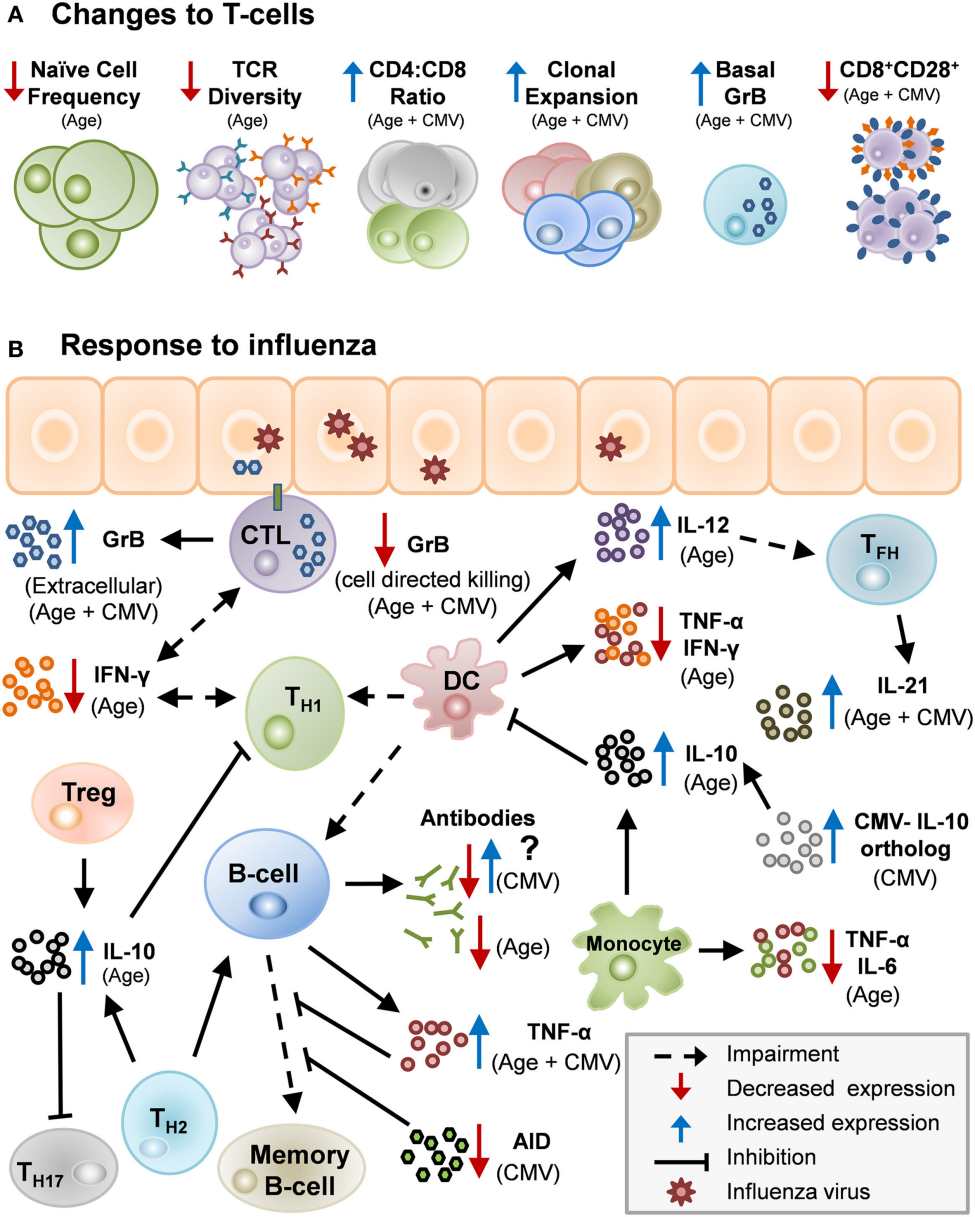
Potential mechanisms by which age and cytomegalovirus (CMV) may cause changes in human immunity. **(A)** Age and CMV can act in unison to impair aspects of the immune system: lower numbers of naïve T-cells, decrease in T-cell receptor (TCR) repertoire, heightened CD4:CD8 ratio, clonal expansion, increased levels of basal granzyme B (GrB) in resting T-cells and decrease in CD8^+^CD28^+^ cells. **(B)** There are several potential mechanisms by which age and CMV may cause changes in human immunity. Influenza infection stimulates Th1/Th2 although impaired in older adults due to diminished antigen-presenting cell function in the elderly. Th1 response with IFN-γ activating memory cytotoxic T-lymphocyte (CTL) which clears virus from the lungs. Age-related changes drive a Th2/regulatory T-cell (Treg) response to infection, and IL-10 production suppresses the CTL response. CMV infection further impairs the response to influenza infection by contributing to age-related impairments and by other mechanisms.

Aging, along with CMV seropositivity, impacts the immune response to influenza infection and vaccination as a result of many interacting mechanisms (Table 1; Figure 1). With the increased risk of influenza-associated morbidity and mortality in the over-65 population, it is critical to take these impairments into consideration when developing the next generation of influenza vaccines.

**Table 1:** Summary of the impact of cytomegalovirus (CMV) in the elderly and resulting influence on the response to influenza infection and vaccination.

Impact of CMV	Impact on immune system	Impact on influenza infection/vaccination	Reference
CMV-encoded IL-10 ortholog	Inhibits dendritic cell function by hindering maturation and functionality	Potentially poor antigen-presenting cell capacity during infection or response to vaccination	(37)
Elevated IL-21	Greater frequency of activated pT_FH_	Associated with improved antibody response to influenza vaccination	(109–112)
Reduction in activation-induced cytidine deaminase	Impaired class switch recombination and somatic hypermutation	Diminished antibody response to influenza vaccination	(54, 55)
Increased TNF-α in B-cells	Causes B-cell activation, systemic inflammation, and reduced function	Poor antibody class switching	(41, 56, 57)
Lower GrB response		Potential impairment of cytotoxic T-lymphocyte response to influenza infection	(63)
Loss of naïve CD4^+^ and CD8^+^ T-cells	Associated with an increase in effector and effector memory CD4^+^ and CD8^+^ T-cells	Potentially reducing the ability to develop a response to new virus antigens	(86)
Elevated number of CD8^+^CD28^−^ and CD4^+^CD28^−^ cells	Reduced ability for cell activation	Associated with poor response to vaccination	(156–160)

## Author Contributions

SM developed the initial outline and draft of the manuscript. GP, GK, and JM revised the manuscript critically for important intellectual content. All the authors read and approved the final manuscript.

## Conflict of Interest Statement

SM, GP, and GK have no conflict of interest to declare. JM has travel support and her Institute has received honoraria for her participation in advisory boards or presentations at meetings from GSK, Sanofi, and Pfizer.

## Abbreviations

AID, activation-induced cytidine deaminase; APC, antigen-presenting cell; BCR, B-cell receptor; CMV, cytomegalovirus; CTL, cytotoxic T-lymphocyte; DC, dendritic cell; EBV, Epstein–Barr virus; GrB, granzyme B; HA, hemagglutinin; M1, matrix 1; MHC, major histocompatibility complex; NA, neuraminidase; NP, nucleoprotein; pH1N1, pandemic H1N1; pT_FH_, peripheral T-follicular helper cell; TCR, T-cell receptor; Treg, regulatory T-cell.
